# P-1289. Extended Stability of Rectal Swab Specimens for Carbapenemase Gene Detection Using the Cepheid Xpert® Carba-R Assay

**DOI:** 10.1093/ofid/ofaf695.1477

**Published:** 2026-01-11

**Authors:** Shannon L Morris, Kailee Cummings, Kimberlee A Musser, Janine Bodnar, Mohammad Khan, Ashley Marcinkiewicz, Spencer Kennedy, nicola Faraci, Christine Jacobsen, Kara Miller, Majie C Foster, Elizabeth Nazarian

**Affiliations:** NYSDOH- Wadsworth Center, Voorheesville, NY; NYSDOH- Wadsworth Center, Voorheesville, NY; Wadsworth Center, New York State Department of Health, Albany, NY; New York State Dept of Health, Albany, New York; NYDOH Wadsworth Center, Troy, New York; NYSDOH Wadsworth Center, Albany, New York; Wadsworth Center, Albany, New York; Wadsworth Center-New York State Dept of Health, Albany, New York; New York State Dept of Health, Albany, New York; Wadsworth Center, Albany, New York; Wadsworth Center, NYSDOH, ALBANY, New York; Wadsworth, Albany, NY

## Abstract

**Background:**

The Wadsworth Center (WC) serves as the Northeast Regional Antimicrobial Resistance (AR) Laboratory, specializing in the identification and characterization of carbapenemase-producing organisms (CPOs). WC performs colonization screenings (CS) from rectal swabs using the FDA-cleared Cepheid Xpert® Carba-R PCR assay, which detects five major carbapenemase genes (*bla*_KPC_, *bla*_NDM_, *bla*_VIM_, *bla*_OXA-48_, and *bla*_IMP_). According to the FDA cleared package insert, rectal swabs collected are stable for up to five days at 15°C–28°C prior to testing. This CS is performed in collaboration with healthcare facilities, infection preventionists, and epidemiologists to screen high-risk patients and assess CPO transmission events.

**Methods:**

WC conducted stability studies to evaluate extended storage times and temperatures, as well as recovery of viable gene-positive organisms. Mock rectal swab specimens were prepared by inoculating sterile swabs with pooled, known-negative fecal samples. Inoculated swabs were dosed with 50 µL of a bacterial suspension with a target gene and stored (-18°C to 35.5°C) across multiple time points (Days 0, 1, 4, 6, 8, 11, 15, 21, 28, and 46/47) to mimic transport conditions. Testing was performed with the Carba-R PCR assay on the GeneXpert® Infinity System. All Carba-R positive samples were also processed for culture and organism isolation.

**Results:**

The results demonstrated that bacterial DNA remained stable across all evaluated temperatures, with all five target genes consistently detected for at least 46 days post-collection. However, if specimens are submitted for culture isolation, viability varies and was determined to be: 6 days when stored frozen, 11 days when refrigerated, and 46 days at room temperature or higher.

**Conclusion:**

These findings demonstrate that rectal swabs collected and tested with the Xpert® Carba-R PCR assay maintain stability and gene detection well beyond the current FDA-cleared specifications. This study will allow our laboratory to extend acceptability criteria and will provide the ability to perform testing when exceptional circumstances delay shipments or testing to provide public health impact by identifying and stopping CPO transmission in healthcare facilities.

**Disclosures:**

All Authors: No reported disclosures

